# Supplementary material for: Development of Bag-1L as a therapeutic target in androgen receptor-dependent prostate cancer
Source: eLife. 2017 Aug 10;6:e27159. doi: 10.7554/eLife.27159 (PMC5629025; doi:10.7554/eLife.27159)
Supplement: Figure 3—source data 1. [file elife-27159-fig3-data1.docx]

**Figure 3-source data 1**

| Protein (Uniprot accession | Structure in complex with protein (uniprote accession | Organism | PDB code and chain | Resolution |
| --- | --- | --- | --- | --- |
| BAG1 (Q99933) | HSPA8 (P11142) | HUMAN | 3FZM_B | 2.3Å |
| BAG1 (Q99933) | HSPA8 (P11142) | HUMAN | 5AQN_F | 2.45Å |
| BAG1 (Q99933) | HSPA8 (P11142) | HUMAN | 5AQG_B | 2.24Å |
| BAG1 (Q99933) | HSPA8 (P11142) | HUMAN | 5AQJ_D | 1.96Å |
| BAG1 (Q99933) | HSPA8 (P11142) | HUMAN | 5AQN_D | 2.45Å |
| BAG1 (Q99933) | HSPA8 (P11142) | HUMAN | 5AQO_F | 2.12Å |
| BAG1 (Q99933) | HSPA8 (P11142) | HUMAN | 5AQM_B | 1.63Å |
| BAG1 (Q99933) | HSPA8 (P11142) | HUMAN | 5AQL_D | 1.69Å |
| BAG1 (Q99933) | HSPA8 (P11142) | HUMAN | 5AQM_D | 1.63Å |
| BAG1 (Q99933) | HSPA8 (P11142) | HUMAN | 3FZK_B | 2.1Å |
| BAG1 (Q99933) | HSPA8 (P11142) | HUMAN | 5AQP_D | 2.08Å |
| BAG1 (Q99933) | HSPA8 (P11142) | HUMAN | 5AQI_D | 1.98Å |
| BAG1 (Q99933) | HSPA8 (P11142) | HUMAN | 5AQI_B | 1.98Å |
| BAG1 (Q99933) | HSPA8 (P19120) | HUMAN | 1HX1_B | 1.9Å |
| BAG1 (Q99933) | HSPA8 (P11142) | HUMAN | 5AQK_B | 2.09Å |
| BAG1 (Q99933) | HSPA8 (P11142) | HUMAN | 5AQR_D | 1.91Å |
| BAG1 (Q99933) | HSPA8 (P11142) | HUMAN | 5AQN_B | 2.45Å |
| BAG1 (Q99933) | HSPA8 (P11142) | HUMAN | 5AQG_D | 2.24Å |
| BAG1 (Q99933) | HSPA8 (P11142) | HUMAN | 5AQS_B | 2Å |
| BAG1 (Q99933) | HSPA8 (P11142) | HUMAN | 5AQH_B | 2Å |
| BAG1 (Q99933) | HSPA8 (P11142) | HUMAN | 5AQF_D | 1.88Å |
| BAG1 (Q99933) | HSPA8 (P11142) | HUMAN | 5AQP_B | 2.08Å |
| BAG1 (Q99933) | HSPA8 (P11142) | HUMAN | 5AQF_B | 1.88Å |
| BAG1 (Q99933) | HSPA8 (P11142) | HUMAN | 5AQJ_F | 1.96Å |
| BAG1 (Q99933) | HSPA8 (P11142) | HUMAN | 5AQJ_B | 1.96Å |
| BAG1 (Q99933) | HSPA8 (P11142) | HUMAN | 3FZF_B | 2.2Å |
| BAG1 (Q99933) | HSPA8 (P11142) | HUMAN | 5AQL_B | 1.69Å |
| BAG1 (Q99933) | HSPA8 (P11142) | HUMAN | 5AQQ_D | 2.72Å |
| BAG1 (Q99933) | HSPA8 (P11142) | HUMAN | 5AQR_F | 1.91Å |
| BAG1 (Q99933) | HSPA8 (P11142) | HUMAN | 5AQO_B | 2.12Å |
| BAG1 (Q99933) | HSPA8 (P11142) | HUMAN | 5AQP_F | 2.08Å |
| BAG1 (Q99933) | HSPA8 (P11142) | HUMAN | 5AQQ_B | 2.72Å |
| BAG1 (Q99933) | HSPA8 (P11142) | HUMAN | 5AQS_D | 2Å |
| BAG1 (Q99933) | HSPA8 (P11142) | HUMAN | 3FZH_B | 2Å |
| BAG1 (Q99933) | HSPA8 (P11142) | HUMAN | 5AQG_F | 2.24Å |
| BAG1 (Q99933) | HSPA8 (P11142) | HUMAN | 3FZL_B | 2.2Å |
| BAG1 (Q99933) | HSPA8 (P11142) | HUMAN | 5AQO_D | 2.12Å |
| BAG1 (Q99933) | HSPA8 (P11142) | HUMAN | 3M3Z_B | 2.1Å |
| BAG1 (Q99933) | HSPA8 (P11142) | HUMAN | 3LDQ_B | 1.9Å |
| BAG1 (Q99933) | HSPA8 (P11142) | HUMAN | 5AQU_B | 1.92Å |
| BAG1 (Q99933) | HSPA8 (P11142) | HUMAN | 5AQQ_F | 2.72Å |
| BAG1 (Q99933) | HSPA8 (P11142) | HUMAN | 5AQR_B | 1.91Å |
| BAG1 (Q99933) | HSPA8 (P11142) | HUMAN | 5AQV_B | 1.75Å |
| BAG1 (Q99933) | HSPA8 (P11142) | HUMAN | 5AQT_B | 1.9Å |
