# Supplementary material for: Development of Bag-1L as a therapeutic target in androgen receptor-dependent prostate cancer
Source: eLife. 2017 Aug 10;6:e27159. doi: 10.7554/eLife.27159 (PMC5629025; doi:10.7554/eLife.27159)
Supplement: Table 1—source data 1. — HSPC: hormone sensitive prostate cancer, CRPC: castration-resistant prostate cancer, PSA: prostate specific antigen, HR: hazard ratio, 95% CI: 95% confidence intervals. a Univariate cox survival model. [file elife-27159-table1-data1.docx]

**Table 1-source data 1**

|  |  | **HR** | **95% CI** | **p-value** |
| --- | --- | --- | --- | --- |
| **HSPC** | **Nuclear AR expression (per 10 H-score, continuous variable)** |  |  |  |
|  | PSA progression | 1.03 | 0.97-1.08 | 0.31^a^ |
|  | Radiological progression | 1.06 | 0.97-1.09 | 0.40^a^ |
|  | Overall survival | 1.04 | 0.98-1.10 | 0.24^a^ |
| **CRPC** | **Nuclear BAG-1 expression (per 10 H-score, continuous variable)** |  |  |  |
|  | PSA progression | 1.01 | 0.92-1.10 | 0.85^a^ |
|  | Radiological progression | 1.01 | 0.92-1.11 | 0.84^a^ |
|  | Overall survival | 0.97 | 0.90-1.05 | 0.45^a^ |
|  | **Nuclear AR expression (per 10 H-score, continuous variable)** |  |  |  |
|  | PSA progression | 0.50 | 0.22-1.13 | 0.10^a^ |
|  | Radiological progression | 1.00 | 0.95-1.05 | 0.98^a^ |
|  | Overall survival | 0.98 | 0.93-1.02 | 0.33^a^ |
