# Supplementary material for: Development of Bag-1L as a therapeutic target in androgen receptor-dependent prostate cancer
Source: eLife. 2017 Aug 10;6:e27159. doi: 10.7554/eLife.27159 (PMC5629025; doi:10.7554/eLife.27159)
Supplement: Table 1—source data 2. — CRPC: castration-resistant prostate cancer, ECOG PS: Eastern Cooperative Oncology Group performance status, IQR: interquartile range, SD: standard deviation, PSA: prostate specific antigen, n: number, pts: patients. at-test from linear regression model of Nuclear Bag-1 H-score at the time of CRPC biopsy bWald test from linear regression model of Nuclear Bag-1 H-score at the time of CRPC biopsy [file elife-27159-table1-data2.docx]

**Table 1-source data 2**

|  |  | **All patients**  **38 pts** | **p-value** |
| --- | --- | --- | --- |
| **At CRPC biopsy** | **Age, yr** |  |  |
|  | Median | 70.0 | 0.19^a^ |
|  | IQR | 65.7-75.0 |  |
|  | **Sites of metastasis, n (%)** |  |  |
|  | Node only | 2 (5) | 0.04^b^ |
|  | Bone only | 29 (76) |  |
|  | Visceral (with/without bone) | 7 (18) |  |
|  | **ECOG PS, n (%)** |  |  |
|  | 0 | 9 (24) | 0.25^b^ |
|  | 1 | 27 (71) |  |
|  | 2 | 2 (5) |  |
|  | **PSA, μg/L** |  |  |
|  | Median | 242.0 | 0.68^a^ |
|  | IQR | 120.0-664.8 |  |
|  | **Hemoglobin, g/L** |  |  |
|  | Mean | 112.8 | 0.55^a^ |
|  | SD | 13.8 |  |
|  | **Alkaline phosphatase, U/L** |  |  |
|  | Median | 144.5 | 0.10^a^ |
|  | IQR | 82.5-347.5 |  |
|  | **Lactate dehydrogenase, U/L** |  |  |
|  | Median | 190.0 | 0.67^a^ |
|  | IQR | 149.3-259.5 |  |
|  | **Albumin, g/L** |  |  |
|  | Mean | 34.1 | 0.02^a^ |
|  | SD | 4.3 |  |
|  | **Treatments prior to/on for CRPC at biopsy, n (%)** |  |  |
|  | Docetaxel | 23 (61) | 0.80^a^ |
|  | Abiraterone | 19 (50) | 0.37^a^ |
|  | Enzalutamide | 3 (8) | 0.18^a^ |
|  | Cabazitaxel | 6 (16) | 0.41^a^ |
|  | **Treatment for CRPC after biopsy, n (%)** |  |  |
|  | Docetaxel | 12 (32) | 0.94^a^ |
|  | Abiraterone | 19 (50) | 0.37^a^ |
|  | Enzalutamide | 6 (16) | 0.82^a^ |
|  | Cabazitaxel | 15 (39) | 0.14^a^ |
